# Room-Temperature H_2_ Gas Sensing Characterization of Graphene-Doped Porous Silicon via a Facile Solution Dropping Method

**DOI:** 10.3390/s17122750

**Published:** 2017-11-28

**Authors:** Nu Si A. Eom, Hong-Baek Cho, Yoseb Song, Woojin Lee, Tohru Sekino, Yong-Ho Choa

**Affiliations:** 1 Department of Fusion Chemical Engineering, Hanyang University, Ansan 15588, Korea; djafntldk@hanmail.net (N.S.A.E.); hongbaek27@gmail.com (H.-B.C.); licanen@nate.com (Y.S.); 2 Process Development Team, Semiconductor R&D Center, Samsung Electronics Co., Ltd., Samsungjeonja-ro 1, Hwaseong, Gyeonggi-do 445-330, Korea; woojina.lee@samsung.com; 3 The Institute of Scientific and Industrial Research, Osaka University, 8-1 Mihogaoka, Ibaraki, Osaka 567-0047, Japan; sekino@sanken.osaka-u.ac.jp

**Keywords:** graphene-doped porous silicon, p-type silicon, hydrogen sensor, sensing mechanism

## Abstract

In this study, a graphene-doped porous silicon (G-doped/p-Si) substrate for low ppm H_2_ gas detection by an inexpensive synthesis route was proposed as a potential noble graphene-based gas sensor material, and to understand the sensing mechanism. The G-doped/p-Si gas sensor was synthesized by a simple capillary force-assisted solution dropping method on p-Si substrates, whose porosity was generated through an electrochemical etching process. G-doped/p-Si was fabricated with various graphene concentrations and exploited as a H_2_ sensor that was operated at room temperature. The sensing mechanism of the sensor with/without graphene decoration on p-Si was proposed to elucidate the synergetic gas sensing effect that is generated from the interface between the graphene and p-type silicon.

## 1. Introduction

Hydrogen gas is widely used as a clean fuel in various industrial fields, and is expected to be the fuel to replace fossil fuels [1]. Since H_2_ is known to be highly colorless, odorless, and explosive at concentrations greater than 4% [2], the ability to detect early hydrogen leakage is prerequisite and demand for a highly sensitive H_2_ gas sensor is increasing. In general, gas sensors are of great interest because of their ability for real time analysis of gaseous chemicals over a wide range of applications. In the vast area of gas sensing, hydrogen sensors are based on metal oxide films that are configured in a chemiresistor mode [3]. Typical metal oxide gas sensors have a high power consumption due to their high working temperatures (200–400 °C) [4]. Micro hotplate of low consumption has also power consumption over ten to hundred mW [5,6]. When compared to other semiconductor gas sensors, a porous silicon (p-Si) gas sensor can be operated at relatively low temperatures, even at room temperature [7]. p-Si is an interesting base material with which to develop a gas sensor due to their unique combination of crystalline structure, high specific surface area, and high surface chemical activity [8]. Various additives have been incorporated into p-Si to enhance its sensing property [9] because the response is dependent on the base device matrix and the influence of catalytic components, like Pd, Pt, and Ru. [10].

Gas sensing is one of the most promising applications for graphene, because the delocalized pi(p) bonds of graphene allow for charge carriers to have zero rest mass and high mobility [11,12], and graphene has high surface-to-volume ratio and high surface chemical activity for enhanced adsorption of gases on the basal surface [13]. Graphene is utilized as a matrix material for the sensor of a general graphene-based gas sensor, which can be easily synthesized by the incorporation of other supportive sensor materials, like metal oxides [14]. The operational principle of such graphene devices is based on changes in their electrical conductivity due to gas molecules that are adsorbed on the graphene surface acting as donors or acceptors, similar to other solid-state sensors [15,16]. However, to the best of our knowledge, there have been no studies that have investigated the gas sensing property describing incorporation of a graphene decoration on a p-Si matrix.

In this study, the sensor properties for low ppm H_2_ gas detection based on graphene-doped porous silicon (G-doped/p-Si) substrate are investigated utilizing graphene as a catalyst material. Graphene doping was performed by a facile, inexpensive synthetic procedure, using solution dropping onto a p-Si substrate with a one-step method of decorating graphene on the high surface area porous media created via an electrochemical etching process. Electrochemical etching is typically a simple, inexpensive procedure for synthesizing p-Si layers [8]. The loaded amount of graphene was varied as a function of graphene concentration, ranging from 0 to 10 mg/mL in an aqueous solution, whose potential as a hydrogen gas sensor was evaluated during operation at room temperature. Drawing on graphene’s intrinsic properties of high mobility and conductivity, attention was focused on exploring the role of the formation of the electrical junction between graphene-to-silicon interfaces for the enhancement of hydrogen gas detection.

## 2. Materials and Methods

### 2.1. Material

Water-dispersible graphene with oxygen functionalization at basal edges was developed by MExplorer Co., Ltd. (Ansan, Korea), the thickness and lateral dimension of the as-received graphene was <5 nm and 2–3 μm, respectively (See Figure S1).

### 2.2. Synthesis of the P-Si Substrate

The synthesis of the high porosity silicon substrate was performed by a technique combining both metal-assisted chemical etching (MacE) and electrochemical etching [17], utilizing a p-type monocrystalline silicon wafer with a 300 μm thickness, <100> orientation and 1–10 Ω resistance. To conduct the MacE process, Pt-catalyst loading on the prepared porous silicon was performed by the sputter process (Quorum Technologies, Q150T ES, East Sussex, UK) utilizing the Pt (purity: 99.99%) target. The domains of the deposited Pt metal particles with a nano-dimension ranging from 30 to 60 nm were formed on the Si substrate after deposition and heat treatment at 650 °C. After cooling to room temperature, the Pt particle-doped silicon wafer was attached to aluminum foil for electrochemical etching. The silicon substrate and Pt electrode were connected to a DC power supply (E3647A, Agilent, Palo Alto, CA, USA) as a working electrode and counter electrode, respectively, which were computer controlled. A current density of 1 mA/cm^2^ was applied for 1h in a mixture solution of 30 wt % H_2_O_2_ and 10 wt % HF. After the chemical etching, the porous silicon substrate was dipped in 10 wt % HF to remove the formed oxide layer from the silicon surface for the electrochemical etching processes, and the samples were then rinsed copiously in DI water and dried at room temperature.

### 2.3. Synthesis of Graphene-Doped Porous Silicon Heterostructure

Doping graphene on the synthesized p-Si substrate was performed by a facile solution drop method using a micro pipet. The 100 μL solution volume as a function of graphene concentration ranging from 0 to 10 mg/mL in an aqueous solution on the p-Si substrate size (5 × 10 mm^2^) was deposited (Figure S2) and dried for 20 min in atmosphere at 100 °C. The morphology of the Pd-doped p-Si was analyzed using a scanning electric microscope (SEM, MIRA3, TESCAN Ltd., Warrendale, PA, USA). A 200 nm thick gold layer for the sensor electrode was evaporated on the top of each p-Si surface to create the electrical contact, except for the rectangle-shaped sensing area (5 × 1 mm^2^) that was located in the middle part on the surface of substrate (Figure S2). The formed electrode and H_2_ sensing properties of the sensor system were measured at room temperature in a glass chamber (Figure S2) and recorded online by a NI PXle-1073 (National Instruments Corporation, Austin, TX, USA). The gas concentrations were controlled by changing the mixing ratio of dry air and H_2_ using mass flow controllers and the total gas flow rate was set at 500 sccm. The experiments were carried out with an applied voltage of 5.0 V. The sensitivity S was used to characterize the sensor performance using the equation S = ∆R/R_a_ = │(R_a_ − R_g_)│/R_a_ × 100%, where R_a_ and R_g_ are the electrical resistance of the sensor under dry air and the concentration of H_2_ gas, respectively.

## 3. Results

**Figure 1:**
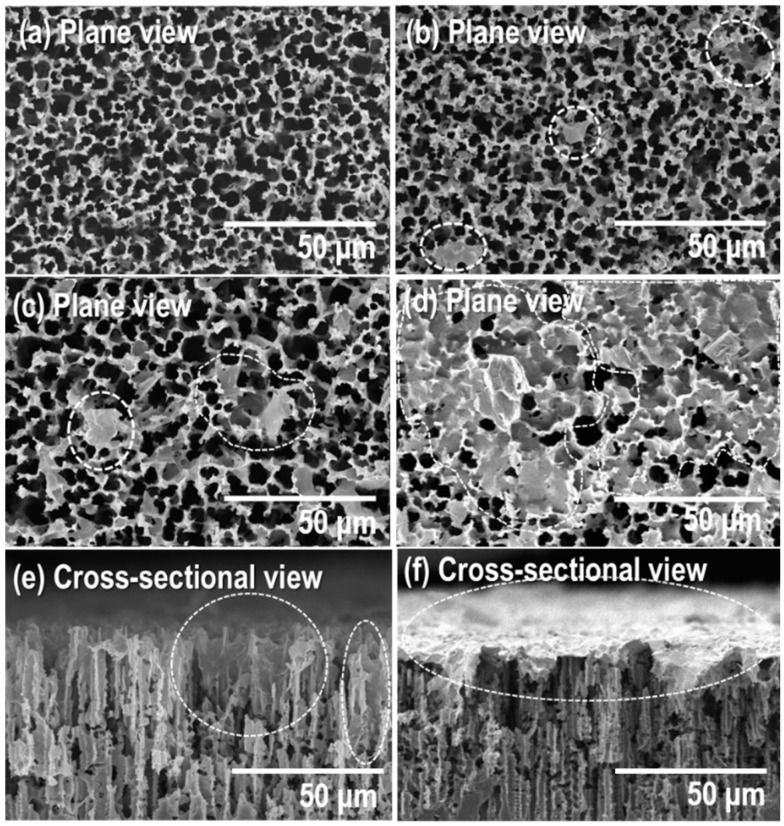
Plane and cross-sectional SEM images of the G-doped/p-Si with (**a**) 0 (**b**) 0.1(**c**,**e**) 1 and (**d**,**f**), 10 mg/mL graphene solution concentrations.

Figure 1 shows the SEM image of the G-doped/p-Si dried after doping 100 uL of the graphene solution on p-Si substrate. The loaded amount of graphene on the p-Si wafer substrate was increased as the concentration of graphene increased from 0 to 10 mL/mg. The quantity of graphene on the surface was predicted drawing on the calculation of solution concentration and volume. Graphene solution 100 uL was deposited on porous silicon substrate (5 × 10 mm^2^). The estimated graphene volume is (a) 0 mg/mL, (b) 0.1 mg/mL (graphene volume: 0.01 mg), (c) 1 mg/mL (graphene volume: 0.1 mg), and (d) 10 mg/mL (graphene volume: 1 mg). The size of the graphene identified from the SEM view (the dotted domains) ranged from several to tens of microns. The external average pore size of the synthesized p-Si is 4.5 μm macroporosity, whose width narrows to meso- and micro-porosity toward a vertical hole depth of approximately 90 μm (see Figure S3), which could be confirmed by the nano-dimensional cracks on the inside wall of the p-Si (see Figure S3 inset). This porous structure enables the decoration of graphene 2–3 μm inside the surface. Upon adding the graphene solution drop-wise to the surface, it diffused into the p-Si by capillary force [18] from the lateral dimension of the as-received graphene. In spite of this, when the concentration of the graphene increases, a larger amount of graphene appears on the external surface of the p-Si (Figure 1a–d), and on the internal porous wall near the silicon surface (Figure 1e). The majority of the external surface was then covered by graphene when the graphene concentration reached 10 mg/mL, as shown in Figure 1f. Thus, the sensing properties can be tuned if a heterojunction created between the decorated graphene-to-silicon interface occurs throughout the extended p-Si surfaces.

**Figure 2:**
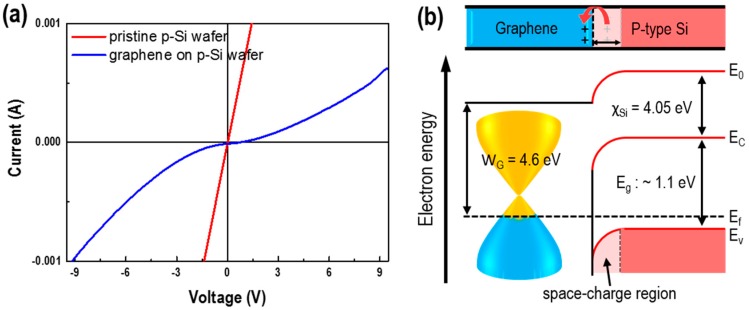
(**a**) Current-Voltage (IV) curve and (**b**) junction schematic of a pristine p-Si wafer and G-doped/p-Si sensors at room temperature.

Figure 2 shows the IV behaviors and energy band diagrams of pristine and graphene-deposited p-Si that confirms the creation of a heterojunction between the p-type Si of the semiconductor and graphene of the semi-metal with deposited graphene on the pristine p-Si. This can be consulted from the report describing the formation of the Schottky barrier at the graphene/silicon interface [19], the partial carriers in p-type silicon tends to move to the graphene (Figure 2a), and, consequently, the energy levels near the silicon surface will bend downward (Figure 2b), facilitating the formation of a space-charge region and built-in electric field near the graphene/silicon interface due to the work function of graphene (4.6 eV) more than p-type silicon. Therefore, the IV curve shows that with and without graphene deposited, p-Si has ohmic and Schottky junctions, respectively.

**Figure 3:**
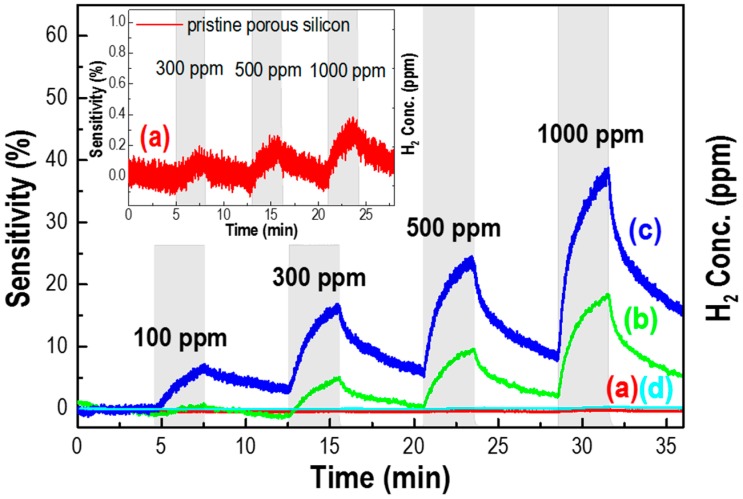
Response of a pristine p-Si wafer (thickness 70 μm) and G-doped/p-Si sensors with (**a**) 0; (**b**) 0.1; (**c**) 1; and, (**d**) 10 mg/mL graphene concentrations to an air-based H_2_ gas operated at room temperature.

Figure 3 shows the H_2_ sensing properties of the G-doped/p-Si as a function of graphene concentration at room temperature. The pristine p-Si wafer has little sensing property for hydrogen molecules with a sensitivity of less than 0.5% under 100 to 1000 ppm H_2_ at a flow rate of 500 sccm (see Figure 3a) and also the inset), whereas the p-Si with graphene has an obvious increase in sensing H_2_ gas, with a concentration ranging from 100 to 1000 ppm (Figure 3b). The sensing property of p-Si with doped graphene increased with an increase in H_2_ gas concentration, as well as graphene solution concentration. Hydrogen gas can be adsorbed on the surface of p-type silicon, where the gas molecules extract holes from the adsorbed surface of silicon that could form ionized hydrogen by the hydrogen redox reaction [19], contributing to a very weak variation of sensitivity, as shown in the inset of Figure 3. Moreover, as described in Figure 2 over G-doped/p-Si, the major carrier (hole) originated from the p-type silicon transfers to graphene and motivates the adsorption of the hydrogen gas molecule. Thus, the G-doped silicon surface enhances the sensing property in comparison to the pristine p-Si substrate, drawing on the type of adsorbed gas molecules on the graphene surface that act as donors or acceptors [20]. On the other hand, for the surface of p-Si with 10 mg/mL doped graphene, the majority of the porosity was covered by graphene (Figure 1f), exhibiting a very low sensitivity that is similar to pristine p-Si (Figure 3d).

The G-doped/p-Si does not have a sensing property because p-Si hinders the diffusion of H_2_ gas to the active site of graphene-silicon, located deep inside the porous wall, and the current flow paths are generated only from the graphene surface. Graphene doped porous silicon has low power consumption value (Mean.: 3.243 mW, Max.: 3.71 mW) due to operation at room temperature in comparison with micro hotplate sensors, which are known for low consumption sensors (250 mW at 400 °C) [5] and (16 and 60 mW in the range from 160 to 390 °C) [6].

**Figure 4:**
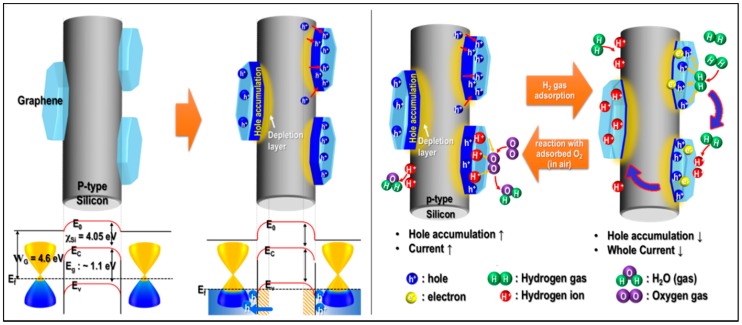
Schematic illustration showing the adsorption and desorption mechanism of H_2_ gas on the surface of doped-graphene on a p-Si wafer.

Figure 4 shows the band diagram between graphene and p-type silicon. Graphene has a lower work function than the p-type silicon 4.6 eV (WG = 4.6 eV), and p-type silicon has an electron affinity of 4.05 eV (χSi) and a band gap of ~1.1 eV (Eg), causing a Schottky junction due to the contact between graphene and p-type silicon [21]. When the junction is generated between graphene and p-type silicon, the major carrier of p-type silicon moves to graphene due to the disparity in work function, forming an electric depletion layer near the p-type silicon and the hole accumulation layer near the graphene [22]. The electric depletion layer was formed over a wide internal surface area of the p-Si substrate with a large specific area. Upon adsorption of hydrogen gas molecules to the surface of G-doped/p-Si, the accumulated holes near the graphene react with hydrogen molecules, which could form ionized hydrogen by the hydrogen redox reaction H_2_ + 2h^+^ → 2H^+^, resulting in a reduction in the carrier density [19]. The conduction of G-doped/p-Si decreased due to the decreased graphene carrier concentration. On the other hand, when the hydrogen gas was removed, the oxygen molecule in air reacts with the formed ionized hydrogen on the graphene and p-type silicon, which increases the hole accumulation layer of graphene and decreases the ionized hydrogen in p-type silicon, resulting in the final conductivity increase of the G-doped/p-Si.

## 4. Conclusions

In this study, a graphene decorated porous silicon (G-doped/p-Si) substrate for low ppm H_2_ gas detection by an inexpensive synthesis route was proposed, where the p-Si substrate with a large specific area was employed as a sensor matrix and graphene as a catalyst material. The H_2_ sensing properties as a function of graphene concentration on the p-Si substrate were analyzed at room temperature. The G-doped/p-Si has an enhanced sensing property in comparison to that of pristine p-Si. The catalytic effect of graphene on the surface of p-Si was elucidated by an enhancement in the carrier transfer to the adsorbed hydrogen gas molecules due to the doped graphene on the p-type silicon. The hierarchical hybrid structure of G-doping on porous silicone shows potential as an extended application of optical and medical sensors, and electronics for storage devices.

## Supplementary Materials

The following are available online at http://www.mdpi.com/1424-8220/17/12/2750/s1, Figure S1: (a) Optical microscope (OM) and (b) TEM images of the as-received graphene, Figure S2: Schematic of sensing system, Figure S3: (a) Surface and (b) cross-sectional morphology of porosity generated on the pristine Si substrate with an average thickness of 90 μm.
