# Supplementary material for: Room-Temperature H2 Gas Sensing Characterization of Graphene-Doped Porous Silicon via a Facile Solution Dropping Method
Source: Sensors (Basel). 2017 Nov 28;17(12):2750. doi: 10.3390/s17122750 (PMC5751383; doi:10.3390/s17122750)
Supplement: Supplementary file 1 [file sensors-17-02750-s001.pdf]

Supplementary Materials: Room-Temperature H_2 Gas Sensing Characterization of Graphene-doped Porous Silicon *via* a Facile Solution Dropping Method

Nu Si A Eom, Hong-Baek Cho, Yoseb Song, Woojin Lee, Tohru Sekino and Yong-Ho Choa

The majority of graphene (except some aggregated domains on the top-right) deposited on the surface of a Si wafer were less than 5 μm in length, specifically 2-3 μm on average (Fig. S1(a)), and their thickness was less than 5 nm(Fig. S1(b)).The graphene was edge oxidized graphene for stability in moisture as the Raman spectra exhibits D band (Fig. S1(c)). Raman mapping also shows that graphene on porous silicon has very low volume. (green: graphene, blue: silicon substrate) (Fig. S1(d)).

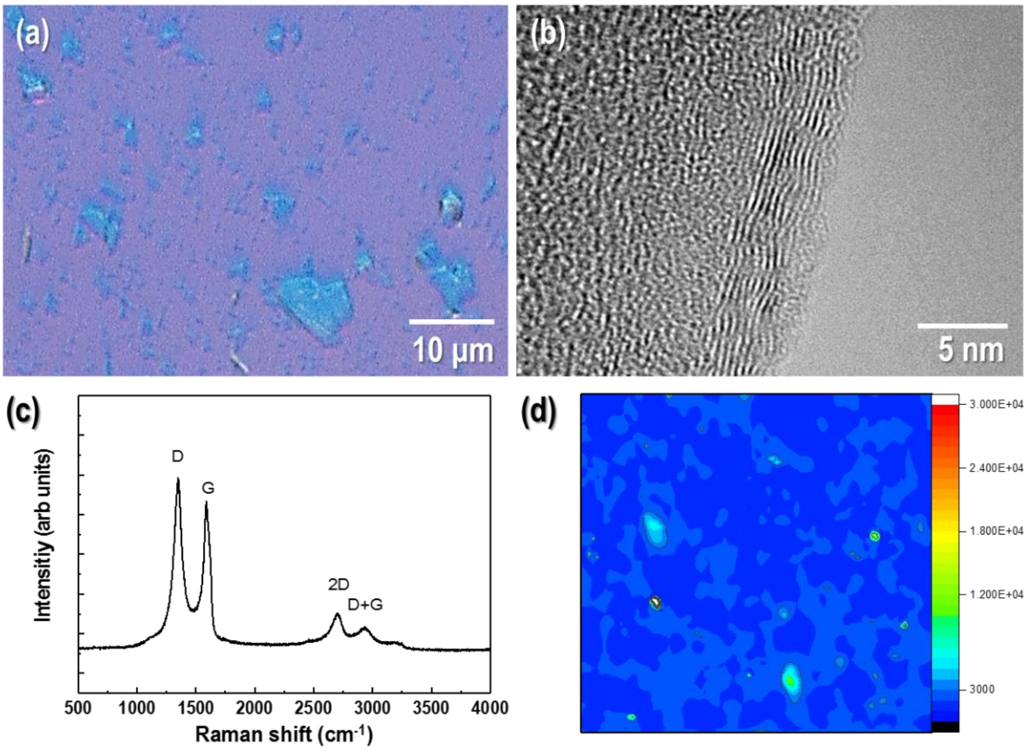

Figure S1. (**a**) Optical microscope (OM); (**b**) TEM images; (**c**) Raman spectra and (**d**) mapping image of the as-received graphene (1 mg/ml (graphene volume: 0.1 mg)).

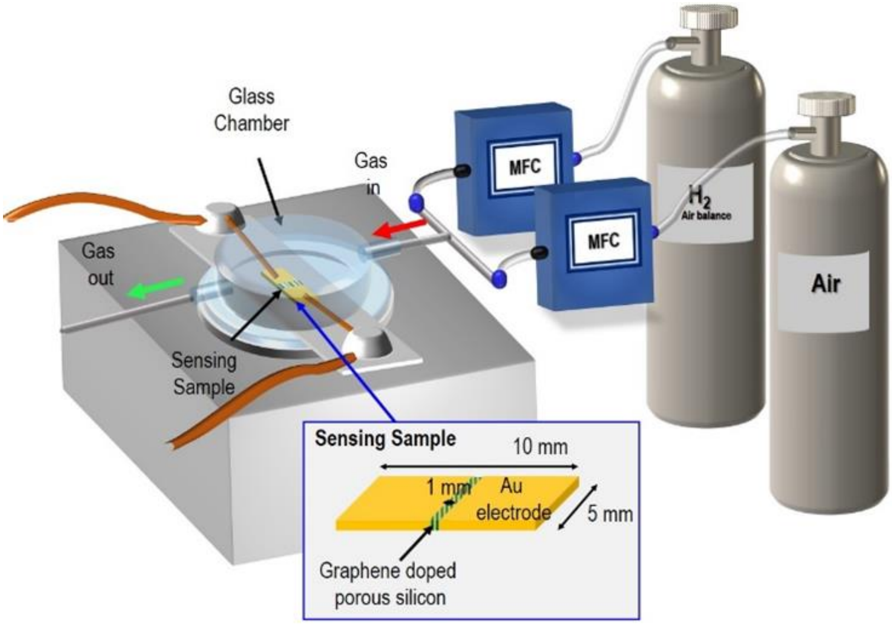

Figure S2. Schematic of sensing system.

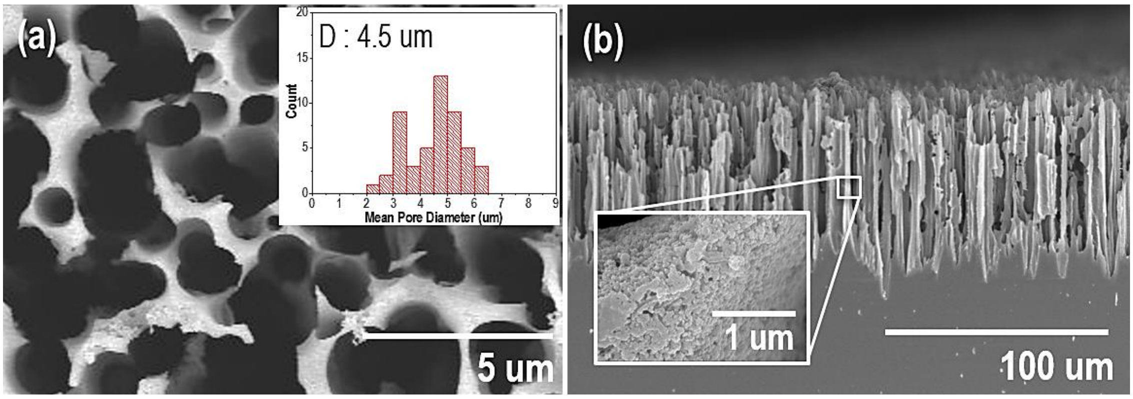

Figure S3. (**a**) Surface and (**b**) cross-sectional morphology of porosity generated on the pristine Si substrate with an average thickness of 90 μm.

The surface image confirms the external pore diameter of Si was 4.5 μm (Fig. S3 (a)) and the cross-sectional image illustrates that the average thickness of the generated vertical pore depth was 90 μm, where the pore tapered towards the inner pores (Fig. S3 (b)).
